# Bilateral high-frequency hearing loss is associated with elevated blood pressure and increased hypertension risk in occupational noise exposed workers

**DOI:** 10.1371/journal.pone.0222135

**Published:** 2019-09-05

**Authors:** Dan Kuang, Yan Yan Yu, Cheng Tu

**Affiliations:** 1 Department of Occupational Disease Prevention and Control, Chengdu Municipal Center for Disease Control and Prevention, Chengdu, Sichuan, China; 2 Department of AIDS/STD Control and Prevention, Chengdu High-tech Center for Disease Control and Prevention, Chengdu, Sichuan, China; Sao Paulo State University (UNESP), BRAZIL

## Abstract

**Objective:**

To investigate the association of bilateral high-frequency hearing loss (BHFHL) with blood pressure and hypertension among occupational noise exposed workers.

**Methods:**

Occupational noise exposed workers were enrolled in 2017 from the occupational diseases survey of Chengdu. BHFHL was classified as normal, mild, or high by the bilateral high-frequency tone average. Linear regression model was used to assess the effects of occupational noise exposure time and BHFHL on blood pressure. Logistic regression model was performed to estimate hypertension risk odds ratios (ORs) associated to occupational noise exposure time and BHFHL.

**Results:**

Increasing years of occupational noise exposure and BHFHL were significantly associated with systolic and diastolic blood pressure increase (all *P*<0.001). The lineal trend was only significant in males, with adjusted ORs for hypertension gradually increasing with increasing years of occupational noise exposure (*P*<0.001). Furthermore, subjects having mild and high BHFHL had a higher hypertension risk of 34% and 281%, respectively (both *P*<0.001). Dose-response relationship between BHFHL and hypertension was found in both males and females.

**Conclusions:**

Occupational noise exposure was positively associated with blood pressure levels and hypertension risk.

## Introduction

Occupational noise is one of the most common occupational hazards in the workplace worldwide [1], with more than 600 million workers exposed to hazardous noise levels [2]. Hearing loss is the primary adverse health effect caused by occupational noise exposure[3]. Occupational noise-induced hearing loss is the most prevalent occupational disease in the United States [4]. Moreover, occupational noise-induced deafness has become the third occupational disease, accounting for 16.7% of the total occupational diseases in China[5]. Thus, hearing loss is a significant occupational health concern in workers exposed to noise[6].

In addition to hearing loss, there are evidences that occupational noise exposure is also associated to other health effects, such as sleep disturbances, psychological stress, cardiovascular diseases, and digestive disorders[7–10]. A number of epidemiologic studies investigated the association of occupational noise exposure with blood pressure and hypertension[11–15]. However, the results are still inconsistent. A Brazilian study reported that noise exposure was independently associated to hypertension, both under low and high exposure in 1729 petrochemical workers[16]. Chen et al. enrolled 1390 occupational noise-exposed workers and 1399 controls, and found a dose-response relationships between noise intensity, years of noise exposure, cumulative noise exposure and the risk of hypertension[12]. Similarly, Chang et al. conducted a prospective cohort study of 578 male workers, and they found a significant exposure-response pattern between the risk of hypertension and the intensity of noise exposure[17]. In contrast, Stokholm et al. performed a 7-year prospective cohort study enrolling 145190 workers, and reported no increased risk of hypertension after noise exposure [15]. Although previous studies investigated the association between occupational noise exposure and hypertension, they are limited by noise exposure assessments. The noise intensity in the workplace could not reflect the actual exposure to noise of each person, which might be the main reason for the inconsistent results.

Occupational noise-induced hearing loss is the change in the perception of the different sound frequencies, and firstly appeared at high frequencies[11, 18, 19]. Furthermore, occupational noise-induced hearing loss is typically bilateral as the symmetric occupational noise exposure[18]. Several studies reported that bilateral high-frequency hearing loss (BHFHL) is associated with cumulative occupational noise exposure, and BHFHL can serve as an early biomarker for the actual personal exposure to occupational noise[11, 18]. Therefore, the aim of this work was to examine whether a dose-response relationship of BHFHL with blood pressure and hypertension is present or not by analyzing the data of a cross-sectional study on Chinese workers.

## Materials and methods

### Subjects

A final number of 21,403 occupational noise exposed workers were enrolled in this study. The exposed workers were recruited in 2017 from a cross-sectional survey of the key occupational diseases in Chengdu, Sichuan Province, China. The key occupational diseases survey is used to estimate the health status of the occupational population in Chengdu by yearly collecting information of all workers exposed to coal dusts, silica dusts, asbestos, benzene, lead, noise, and Brucella. The survey consists of an interview and medical examination. In the interview, various basic information, occupational history, and health-related questions such as age, gender, exposure history to occupational hazards, seniority, and diseases symptoms, are asked by trained interviewers. In the medical examination, various anthropometric and physiological measurements such as blood pressure, routine blood and urine tests, and audiometric testing were performed by trained medical personnel. This cross-sectional survey enrolled a total of 47,992 workers under occupational noise exposure with an intensity >80 dB (A) (L_EX, 8h_). The workers without enough information about blood pressure and bilateral high-frequency tone average (BHFTA), or with chronic diseases such as cancer, cardiovascular disease, diabetes, liver and kidney diseases were excluded. More than 50% of them (26486) were excluded because BHFTA data were not available. The reason was that the medical examiners did not accurately calculate the BHFTA in the physical examination reports as the old audiometric testing devices did not automatically calculate the data. These workers, which did not have the data of BHFTA, came from 1107 factories, and all of the occupational noise exposure workers came from the 1107 factories was not included in this study. Subjects who had reported a diagnosis of hypertension or use of anti-hypertensive medication before they entered into the job which had the occupational noise exposure were also excluded. Finally, a total of 21,403 participants with a median age of 40 years (25^th^ percentile to 75^th^ percentile of the age (P25-P75): 30–46 years)were considered eligible, divided into 15,193 males (median age 38 years, P25-P75: 29–46 years) and 6210 females (median age 41 years, P25-P75: 33-45years). After the occupational health check-up, the prevalence of hypertension in included subjects and exclude subjects was 5.9% (1255/21403) and 6.2% (1650/26589) respectively. No significant difference between excluded and included subjects was observed in prevalence of hypertension (*P* = 0.118>0.05). Before the interview and medical examination, all the participants had read the informed consent form.Written consents were received from all participants, and this work was approved by the Research Ethics Committees of Chengdu Center for Disease Prevention and Control, and conformed to the ethical guidelines of the 1975 Declaration of Helsinki.

### Blood pressure measurement and hypertension definition

Trained medical personnel measured the systolic blood pressure (SBP) and diastolic blood pressure (DBP) of the workers who were free from the occupational noise exposure and at least rest 12 hours following a standard protocol. Blood pressure was measured using a mercury sphygmomanometer on individuals in the sitting position after more than 15-min rest in the examination room. SBP and DBP were recorded as the average of three repeated measurements collected at1-min intervals. Hypertension was defined as SBP ≥ 140 mmHg and/or DBP ≥ 90 mmHg.

### Audiometric testing

Participants were interviewed by trained health technicians to collect detailed hearing-related information including ear illness, toxic drugsuse, poisoning and infectionhistory, and occupational noise exposure history. Audiometric testing was conducted using different audiometers in the sound-isolating room in different hospitals by trained health technicians.The audiometric testing devices in each health examination institution had been calibrated in National Institute of Measurement and Testing Technology, and the error of each device was controlled less than 3%. The workers were free from the occupational noise exposure and at least rest 12 hours, then air conduction hearing thresholds were measured in each ear using a pure tone at seven frequencies (0.5, 1, 2, 3, 4, 6, and 8 kHz) over an intensity range of 10 to 120 decibels hearing level (dB HL). BHFTA was calculated using the arithmetic mean of hearing thresholds at 3, 4, and 6 kHz in both the right and left ears. Normal hearing was defined according to BHFTA < 25 dB. BHFHL was defined as mild when BHFTA was≥ 25 to < 40 dB, while it was defined as high when BHFTA ≥ 40 dB.

### Statistical analysis

All statistical analyses were performed using SPSS 21.0 software (SPSS, Chicago, IL). Categorical variables were expressed as frequencies (%) and compared by Chi-square analysis. Continuous variables were expressed as mean ± standard deviation (SD) in normally distributed data or medians (interquartile ranges) in skewed parameters. Blood pressure was evaluated by linear trend using the median value of occupational noise exposure time and BHFTA as an ordinal variable. A multiple logistic regression model was performed to assess the association between BHFHL and risk of hypertension, with appropriate adjustments for covariates including age, gender, years of occupational noise exposure, living region, enterprise size and economic type. Odds ratio (OR) and its 95% confidence interval (CI) were calculated for the risk of hypertension. Trends for risk of hypertension were assessed using the median value of BHFTA as a continuous variable in the same model. A two-side *P* value <0.05 was considered statistically significant.

## Results

### Study population characteristics

BHFHL prevalence among the enrolled 21,403 individuals was 7.0%. The general characteristics of the participants according to different degrees of BHFTA are summarized in Table 1.Individuals with BHFHL were more likely older, males, living in the second and third ring counties (the city of Chengdu has 22 counties, and the 22 counties are divided into three rings, which included a central city zone (6 counties), the second ring (7 counties), and the third ring (9 counties) according to the distance from the central city zone), and they were working in small and micro enterprises compared with those with a normal hearing level. The prevalence of BHFHL increased stepwise across years of occupational noise exposure (*P*<0.05). Hypertension prevalence was 5.2%, 12.4% and 25.1% in normal hearing, mild and high BHFHL, respectively.

**Table 1:** Basic characteristics of workers exposed to occupational noise by BHFTA levels.

Variables	BHFTA (dB)			P value
	≤25 (n = 19902)	26–39 (n = 1250)	≥40 (n = 251)	
**Age (years)**	39 (30–45)	46 (41–50)	46 (41–51)	<0.001
**Sex, n (%)**				<0.001
** Male**	13911 (69.9)	1051 (84.1)	231 (92.0)	
** Female**	5991 (30.1)	199 (15.9)	20 (8.0)	
**Occupational noise exposure time (years)**				<0.001
** ≤1**	5855 (29.4)	259 (20.7)	15 (9.1)	
** ~3**	5271 (26.5)	281 (22.5)	32 (19.4)	
** ~6**	4209 (21.1)	261 (20.9)	35 (21.2)	
** ~9**	2076 (10.4)	161 (12.9)	20 (12.1)	
**>9**	2491 (12.5)	287 (23.0)	63 (38.2)	
**Hypertension, n (%)**	1037 (5.2)	155 (12.4)	63 (25.1)	<0.001
**Location, n (%)**				<0.001
** First ring**	3840 (19.3)	251 (20.1)	16 (6.4)	
** Second ring**	9587 (48.2)	673 (53.8)	128 (51.0)	
** Third ring**	6475 (32.5)	326 (26.1)	107 (42.6)	
**Enterprise size, n (%)**				0.013
** Large**	3364 (16.9)	188 (15.0)	24 (9.6)	
** Medium**	5442 (27.3)	356 (28.5)	64 (25.5)	
** Small**	9288 (46.7)	599 (47.9)	140 (55.8)	
** Micro**	1808 (9.1)	107 (8.6)	23 (9.2)	
**Economic type, n (%)**				0.410
** Public**	2633 (13.2)	170 (13.6)	31 (12.4)	
** Private**	17131 (86.1)	1077 (86.2)	218 (86.9)	
** Other**	138 (0.7)	3 (0.2)	2 (0.8)	

BHFTA: bilateral high-frequency tone average.

### Occupational noise exposure time, blood pressure and hypertension risk

**Fig 1:**
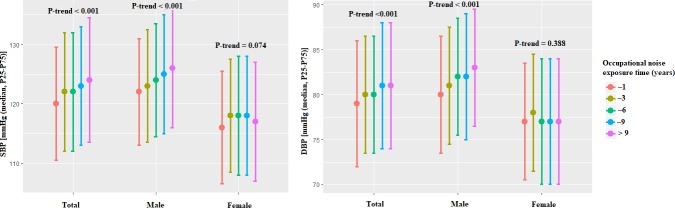
Levels of SBP and DBP in workers by occupational noise exposure time.

SBP and DBP of the people under different occupational noise exposure time are shown in Fig 1. The median levels of SBP and DBP increased stepwise across the occupational noise exposure time. Increasing years of occupational noise exposure were significantly associated with increased SBP and DBP (both *P*<0.001). Stratified analyses by gender were conducted, and significant linear trends were also found in males (both *P*<0.001). However, no significant relationship was found between occupational noise exposure time and blood pressure in females.

The association between occupational noise exposure time and hypertension are shown in Table 2. As shown in this table, increasing years of occupational noise exposure were independently associated with an elevated risk of hypertension after adjustment of age, sex, enterprise location, size and economic type(all *P*<0.05). However, subgroup analyses revealed that the association between the occupational noise exposure time and hypertension was more evident in males (both *P*<0.05), but not in females after multivariable adjustment.

**Table 2:** Adjusted ORs (95% CIs) for hypertension by occupational noise exposure time.

Occupational noise exposure time (years)	Case/participants	OR (95%CIs)		
		Model 1	Model 2	Model 3
**Total**				
** ≤1**	214/6129	1.00 (Ref.)	1.00 (Ref.)	1.00 (Ref.)
** ~3**	278/5584	1.45 (1.21–1.74)	1.25 (1.04–1.51)	1.24 (1.03–1.49)
** ~6**	276/4505	1.80 (1.50–2.17)	1.28 (1.06–1.54)	1.25 (1.04–1.52)
** ~9**	179/2257	2.38 (1.94–2.92)	1.45 (1.18–1.79)	1.45 (1.17–1.80)
**>9**	294/2841	3.19 (2.66–3.83)	1.56 (1.29–1.89)	1.52 (1.25–1.84)
**P-trend**		<0.001	<0.001	<0.001
**Male**				
** ≤1**	166/4310	1.00 (Ref.)	1.00 (Ref.)	1.00 (Ref.)
** ~3**	220/3882	1.5 (1.22–1.84)	1.27 (1.03–1.57)	1.27 (1.03–1.57)
** ~6**	225/3148	1.92 (1.56–2.36)	1.34 (1.08–1.66)	1.33 (1.08–1.65)
** ~9**	158/1644	2.65 (2.12–3.33)	1.61 (1.27–2.03)	1.62 (1.28–2.05)
**>9**	253/2131	3.36 (2.74–4.12)	1.63 (1.32–2.01)	1.64 (1.33–2.04)
**P-trend**		<0.001	<0.001	<0.001
**Female**				
** ≤1**	48/1819	1.00 (Ref.)	1.00 (Ref.)	1.00 (Ref.)
** ~3**	58/1702	1.30 (0.88–1.92)	1.20 (0.81–1.78)	1.20 (0.81–1.78)
** ~6**	51/1357	1.44 (0.97–2.15)	1.09 (0.72–1.63)	1.07 (0.71–1.61)
** ~9**	21/613	1.31 (0.78–2.2)	0.91 (0.54–1.54)	0.94 (0.55–1.61)
**>9**	41/710	2.26 (1.48–3.46)	1.37 (0.89–2.12)	1.43 (0.92–2.23)
**P-trend**		0.001	0.398	0.300

BHFTA: bilateral high-frequency tone average; Model 1 was bivariable analysis; Model 2 adjusted for age and sex; Model 3 adjusted for age, sex, enterprise location, size and economic type.

### BHFHL, blood pressure and hypertension risk

**Fig 2:**
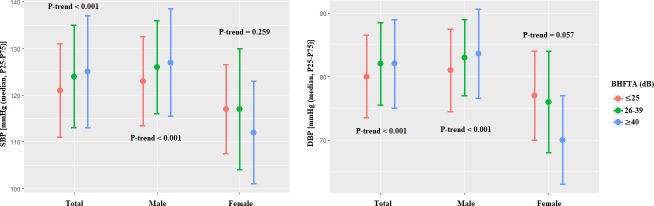
Levels of SBP and DBP in workers by BHFTA levels.

SBP and DBP of the individuals with different BHFTAare shown in Fig 2. SBP median level was 121 (20, interquartile range) mm Hg in the normal hearing group, 124 (22) mm Hg in the mild BHFHL group, and 125 (24) mm Hg in the high BHFHL group. A strong dose-response relationships of BHFTA with SBP and DBP was observed (both *P*<0.05). Further subgroup analyses by gender revealed that dose-response relationships were more pronounced in males (both *P*<0.05), but not in females.

Table 3 shows the hypertension risk in subjects with BHFHL. Compared with the normal hearing group, the individuals with mild BHFHL and high BHFHL had a risk of hypertension with an OR of 1.34 (95%CI: 1.11–1.62) and 3.81 (95%CI: 2.65–5.48), respectively, after adjusting for age, gender, living location, enterprise size and economic type. Stratified analysis by gender showed a dose-response relationship between BHFHL and hypertension in both males and females (both *P*<0.05).

**Table 3:** Adjusted ORs (95% CIs) for hypertension by BHFTA levels.

BHFTA(dB)	Case/participants	OR (95%CIs)		
		Model 1	Model 2	Model 3
**Total**				
** ≤25**	1037/19902	1.00 (Ref.)	1.00 (Ref.)	1.00 (Ref.)
** 26–39**	155/1250	2.58 (2.15–3.08)	1.37 (1.13–1.65)	1.34 (1.11–1.62)
** ≥40**	63/251	6.10 (4.55–8.16)	3.21 (2.36–4.37)	3.81 (2.65–5.48)
**P-trend**		<0.001	<0.001	<0.001
**Male**				
** ≤25**	838/13911	1.00 (Ref.)	1.00 (Ref.)	1.00 (Ref.)
** 26–39**	137/1051	2.34 (1.93–2.84)	1.31 (1.07–1.61)	1.29 (1.06–1.58)
** ≥40**	61/231	5.60 (4.14–7.56)	3.23 (2.36–4.43)	3.39 (2.47–4.66)
**P-trend**		<0.001	<0.001	<0.001
**Female**				
** ≤25**	199/5991	1.00 (Ref.)	1.00 (Ref.)	1.00 (Ref.)
** 26–39**	18/199	2.89 (1.75–4.79)	1.98 (1.18–3.33)	2.01 (1.19–3.4)
** ≥40**	2/20	3.23 (0.75–14.03)	3.01 (0.67–13.5)	3.13 (0.69–14.11)
**P-trend**		<0.001	0.003	0.003

BHFTA: bilateral high-frequency tone average; Model 1 was bivariable analysis; Model 2 adjusted for age and sex; Model 3 adjusted for age, sex, enterprise location, size and economic type.

## Discussion

In this study, BHFHL were used to assess occupational noise exposure, and the results indicated workers having mild and high BHFHL had a higher hypertension risk of 34% and 281%, respectively. In addition, dose-response relationship was found between BHFHL and hypertension in both males and females.

Our results indicated that individuals with BHFHL were more likely older, males, living in the second and third circle counties, and they were working in small and micro enterprises compared with those with a normal hearing level. This result might be explained in following aspects. First, the older the age, the longer the noise exposure. Exposure to high levels of noise was harmful to workers’ hearing and with accumulated effect as time proceeded. Moreover, the older age might result in the loss of neuron and its density, and the decrease of cochlear blood, which made it more vulnerable to noise damage. Second, the noise intensity of the workplace where males and females were involved was significantly different. Male workers were usually exposed to higher noise intensity in their workplace, compared with female workers. Third, the workers living in the second and third circle counties might spend more time on transportation as the far distance from work to home, which might cause more traffic noise exposure. Fourth, the factories in the second and third circle counties were usually small and micro enterprises, which were weakness in occupational health management, did not control the high intensity noise workplaces, and did not offer effective hearing protection equipments and urge the workers to use the hearing protection equipments, such as earplugs, in the noise workplace.

Numerous previous studies investigated the association of noise exposure with blood pressure and hypertension[20–25]. However, the results are inconsistent. A cross-sectional study (n = 2789) based on occupational noise-exposed workers and non-noise-exposed subjects found that occupational noise intensity and time were associated with higher levels of SBP, DBP, and hypertension risk[12]. de Souza TC et al. also observed a significant association between occupational noise intensity and hypertension among 1729 petrochemical workers[16]. Among 578 male workers in a cohort study, Chang et al. showed that prolonged exposure to noise could increase blood pressure levels and a significant exposure-response pattern was found between noise exposure and hypertension risk [17]. A meta-analysis of 12 prospective studies conducted by Skogstad et al. also reported that occupational noise exposure is strongly associated with hypertension[26]. These results were consistent with our study which showed increased occupational noise exposure time association with increased hypertension risk and higher blood pressure. However, a 7-year prospective cohort study (n = 14519) conducted by Stokholm et al. revealed no increased risk of hypertension under anoise exposure at 80–90 dB[15]. Similarly, Kolstad et al. showed no increase of hypertension risk of individuals under anoise exposure above 80 dB on normal working days by following up 100000 blue collar industrial workers[22]. Moreover, the NHANES study that included 4548 participants reported no significant evidence to support the association of occupational noise exposure with blood pressure and hypertension risk[11]. The different results among the above studies may be explained in various ways. One potential reason might be that noise density in working place or noise exposure time could not reflect the accurate personal noise exposure. Noise density in working place, noise exposure time, and earplug wearing might account for the different assessment of noise exposure levels. Another potential reason might be that cross-sectional studies fail to determine temporal or causative relationship of noise exposure with blood pressure and hypertension. As they did not calculate the baseline blood pressure of each individual, it is difficult to discriminate whether hypertension occurred after occupational noise exposure.

In order to exactly evaluate the personal occupational noise exposure, several studies considered BHFHL as a good biomarker because it is associated with intensity and duration of personal noise exposure. A cross-sectional study involving790 aircraft-manufacturing workers showed that workers having high frequency (4 and 6 kHz) hearing loss have higher risk of hypertension[27]. However, SBP and DBP are not significantly different among the hearing loss people in that study. Ni CH et al. found that SBP and DBP in the high frequency hearing loss group are significantly higher than those in the normal hearing group among 618 noise exposed workers[28]. Another study reported that hearing loss at high frequency (4 kHz) is significantly associated with mean blood pressure and hypertension among 119 black men workers[29]. Our findings were consistent with the above articles regarding BHFHL association with higher SBP, DBP, and hypertension risk. The most significant results of this study were the dose-response relationships of BHFHL with blood pressure and hypertension risk. However, the NHANES study including 4548 participants reported no significant linear trends of BHFTA with the increase of blood pressure and hypertension risk. The following differences between the NHANES study and our research might explain the discordant results. First, the participants in our study were the workers whose occupational noise exposure was more than 80 dB according to the report of their workplace, while the NHANES study was based on self-reports of the enrolled individuals regarding their occupational noise exposure. Second, the prevalence of hypertension was 29.7% and 5.9% in the NHANES study and in our study, respectively. Other factors such as age, ethnicity, healthy worker effect, and life habits rather than noise exposure might influence the occurrence of hypertension.

Interestingly, our results showed that the dose-response association between occupational noise exposure time and hypertension risk was more pronounced male workers. Our results partly supported the study by Wang et al., in which hypertension risk was more strongly linked to occupational noise exposure time in males[30]. The potential reason accounting for the gender difference might be that the noise intensity of the workplace where males and females were involved was significantly different. Male workers are usually exposed to higher noise intensity in their workplace, compared with female workers. However, dose-response relationships were found between BHFHL and hypertension in both males and females. This further confirmed BHFHL was a good biomarker, which could reflect the cumulative personal noise exposure level.

This study contains several strengths. First, the present study enrolled more than 20000 workers; evidence based on this large sample size is more powerful and convincing. Second, two methods were used to evaluate occupational noise exposure: noise exposure time and BHFHL, which could strengthen the effectiveness and credibility of the results by mutual confirmation. Third, subgroup analysis considering the gender was performed to avoid potential confounders. However, some limitations are also present in this study. First, the present study was based on a cross-sectional survey, which could not reveal the causal relationship and properly assess the associations. A prospective cohort study is needed to validate the results in the future. Second, although some confounders such as age, gender, enterprise location, size and economic type, were adjusted in the present study, some individual cardiovascular risk factors, such as BMI, cigarette smoking, alcohol consumption, and psychological risk factors, were not considered in the multivariable analysis. Third, this cross-sectional survey enrolled a total of 47,992 workers under occupational noise exposure, and more than 50% of them were excluded because no enough information were available regarding blood pressure or BHFTA. The excluded workers might induce bias to the present results. Fourth, we did not collect the data of noise intensity in the working place, earplug usage and other non-occupational noise exposure of workers, such as the usage of headphones in the way to work and back home, which were very important for hearing loss. In our future research, we will pay more attention to collect the above mentioned data, which could make our research more convincing.

## Conclusions

The present study suggested that occupational noise exposure was positively associated with blood pressure levels and hypertension risk. A dose-response relationship was found between BHFHL and hypertension risk. Further large scale prospective cohort studies are needed to show the cause of this association.

## Supporting information

S1 Table The raw data needed to replicate the findings of this study. (CSV)

S2 Table Survey questions of this study. (DOCX)
