# Supplementary material for: Bilateral high-frequency hearing loss is associated with elevated blood pressure and increased hypertension risk in occupational noise exposed workers
Source: PLoS One. 2019 Sep 5;14(9):e0222135. doi: 10.1371/journal.pone.0222135 (PMC6728038; doi:10.1371/journal.pone.0222135)
Supplement: S2 Table — (DOCX) [file pone.0222135.s002.docx]

**成都市重点职业病监测项目噪声作业工人调查表**

您好！我们是成都市重点职业病监测调查组的成员，这次调查主要想了解成都市噪声作业劳动者的基本特征和健康信息，为开展针对性的职业卫生干预提出切实可行的对策和建议。希望您能接受我们的访问，配合我们共同做好这次调查，对于您提供的信息，我们不会透露给任何无关的人员和机构，谢谢！

一、企业基本信息（由企业提供，职业健康检查机构填报）

1、用人单位名称：___________________ 2、地址：________________________

3、所属行业：________________ 4、企业规模：□大型 □中型 □小型 □微型

5、经济类型：□国有/集体 □私有 □其他

二、劳动者基本信息（询问劳动者，职业健康检查机构填报）

1、姓名：___________2、身份证号：□□□□□□□□□□□□□□□□□□

3、联系电话：___________________

4、体检类型：□岗前体检 □在岗体检 □离岗体检

5、性别：□男 □女 6、出生日期：□□□□-□□-□□

7、总工龄：□□年-□□月 8、接触职业病危害因素名称：____________

9、职业危害接触工龄：□□年-□□月

10、接触所监测危害因素工龄：□□年-□□月

11、既往疾病史：______________________________________________________

三、职业健康检查信息（职业健康检查机构填报）

1、身高_______________cm 2、体重______________________

3、收缩压：________mmHg 4、舒张压：__________________

5、心电图结果：_____________________________

6、噪声双耳高频平均听阈（校正值）：__________dB

7、体检结论：_______________________________________________________

调查单位：________调查人姓名：_______联系电话：_____调查日期：________

**Survey questions of noise workers in the cross-sectional survey of the key occupational diseases in Chengdu**

We are members of the cross-sectional survey of the key occupational diseases in Chengdu. This survey wants to collect the basic characteristics and health information of noise-exposed workers in Chengdu, and to put forward feasible countermeasures and suggestions for carrying out occupational health intervention. I hope you can accept our visit and cooperate with us to do this survey well. We will not disclose any irrelevant personnel and institutions to the information you provide. Thank you.

一、The information of enterprise

1、Name of enterprise：___________________

2、Address：___________________________

3、Industry：_______________________

4、Enterprise size：□Large □Medium □Small □Micro

5、Economic type：□Public □Private □Other

二、The information of workers

1、Name：___________ 2、ID number：□□□□□□□□□□□□□□□□□□

3、Telephone number：___________________

4、Health examination type：□Pre-job □On-job □Post-job

5、sex：□male □female 6、Date of birth：□□□□-□□-□□

7、Work age：□□year-□□month

8、Names of occupational hazards exposed：___________________

9、Occupational hazards exposure time：□□year-□□month

10、Occupational noise exposure time：□□year-□□month

11、History of past illness：_______________________________________________

三、The information of health examination

1、Height:_______________cm

2、Weight:______________________kg

3、Systolic blood pressure：________mmHg

4、Diastolic blood pressure：________mmHg

5、ECG results：_____________________________

6、Bilateral high-frequency tone average：__________dB

7、Conclusions of health examination：_____________________________________

Investigation unit：__________ Investigator：___________

Telephone：__________ Date of survey：________
